# On trial outcomes measured at baseline and after follow-up

**DOI:** 10.1177/14653125211037353

**Published:** 2021-08-24

**Authors:** Spyridon N Papageorgiou

**Affiliations:** Clinic of Orthodontics and Pediatric Dentistry, Center of Dental Medicine, University of Zurich, Zurich, Switzerland

## Theoretical scenario

In this piece, I discuss the hypothetical clinical trial comparing two different methods for the treatment of anterior open bite in adolescent patients. This is based on a recently published trial (Aliaga-Del Castillo et al., 2021), but the sample size has been doubled (by naïve multiplication) from the initially limited sample of 49 patients that underwent randomisation to a sample of 100 patients and has been analysed independently from the original paper.

In this hypothetical trial, patients aged 7–11 years with an anterior open bite between the upper and lower central incisor of at least 1 mm are included and randomised on a 1:1 allocation ratio into two groups. The first group is treated with metal spurs bonded on the palatal / lingual side of the upper / lower incisors combined with posterior build-ups from light-cured orthodontic resin. The second group is treated with the same metallic spurs bonded on the upper / lower incisors, but without the use of posterior build-ups. Intraoral scans of the dentition of all patients are performed before treatment (T1) and after 12 months of treatment (T2) and various dental measurements are taken digitally: overbite; maxillary central incisor crown height (Mx1H); maxillary first molar crown height (Mx6H); and maxillary dental arch perimeter (MxPeri). The authors check first for baseline (T1) differences between groups for each of the outcomes to ascertain similarity between groups and then check for differences due to the treatments administered from the end-of-study values (T2) with Student’s t-test for independent samples with an alpha of 5% (Table 1).

**Table 1.** Descriptive statistics for the trial’s outcomes together with tests for between-group differences.

	G	T1	T2	T2–T1	P_G1–G2_ at T1	P_G1–G2_ at T2
Overbite	G1	–4.0 ± 1.4	0.2 ± 1.6	4.2 ± 1.8	0.65	0.91
	G2	–4.2 ± 2.2	0.2 ± 1.9	4.4 ± 2.0		
Maxillary 1 clinical crown height	G1	8.1 ± 1.4	9.3 ± 0.7	1.2 ± 1.0	0.89	0.15
	G2	8.1 ± 1.3	9.1 ± 0.9	1.0 ± 0.8		
Maxillary 6 clinical crown height	G1	3.4 ± 0.5	3.5 ± 0.4	0.2 ± 0.3	0.04	0.08
	G2	3.1 ± 0.6	3.3 ± 0.6	0.2 ± 0.3		
Maxillary arch perimeter	G1	76.0 ± 3.7	75.5 ± 3.4	–0.5 ± 1.2	0.08	0.07
	G2	77.3 ± 3.4	76.7 ± 3.1	–0.6 ± 1.7		

Values are given as mean ± SD.

G, Group; Mx1H, maxillary central incisor crown height; Mx6H, maxillary first molar crown height; MxPeri, maxillary dental arch perimeter; SD, standard deviation.

## Which of the following statements are correct, if any?

(A) The authors checked for baseline (T1) differences between the randomised groups and concluded that for one of the variables (Mx6H) the randomisation did not work as expected, as t-test gave a *P* < 0.05.
(B) Since baseline (T1) testing for the variable mentioned above (Mx6H) gave a *P* < 0.05 the authors should analyse for this variable any differences between groups using the before-and-after (T2–T1) increment and not the end-of-study values (T2).
(C) Analysing differences between groups with either option for expressing the variable (before-and-after increment [T2–T1] or the end-of-study values [T2]) will give similar results in terms of effect magnitude, *P* values and precision.

## Discussion

Randomisation as a procedure ensures that the formed groups are comparable ‘on average’ for all measured and unmeasured covariates (or better, that the distribution of these baseline covariates is random in both groups). However, random variation of these distributions leading to random baseline imbalance between groups is not unheard of—especially in clinical trials with small samples of recruited patients. This is something that should not necessarily disquiet trialists and eventually it is up to them to know if the randomisation method chosen was valid and applied correctly or was circumvented in any way. However, statistical tests of baseline homogeneity of the randomised groups according to any covariate is a practice that is philosophically unsound, of no practical value and potentially misleading (Altman, 1985; Senn, 1994). The approach performed by the authors is both flawed and wrongly interpreted, since not finding a *P* < 0.05 does not necessarily mean that no imbalance exists. Therefore statement (A) is wrong.

Instead, a potentially more appropriate approach would be for trialists to identify a priori in the trial protocol any potential covariates that might be associated with the outcome of interest and control for them statistically providing adjusted estimates of treatment effects. However, this is not what the authors suggest they do and therefore statement (B) is also wrong.

The author’s choice of analysing the trial’s outcomes by using the before-and-after increment (T2–T1) needs to be adequately discussed. In fact, when an outcome is not measured only after follow-up (e.g. the incidence of death), but is measured both at baseline and after a certain follow-up period, then three (at least) options exist for analysing these data: (1) analysing the end-of-study values (here T2); (2) analysing the before-and-after increment (T2–T1 or T1–T2, respectively); or (3) analysing the end-of-study values (T2) adjusting for baseline values (T1). The results of these three analyses are given in Table 2 using linear regression modelling.

**Table 2.** Statistical analysis (linear regression) for three ways of handling the trial’s outcomes.

Variable		End-of-study value				After-minus-before increment				End-of-study value; adjusted for baseline			
		b	SE	95% CI	*P*	b	SE	95% CI	*P*	b	SE	95% CI	*P*
Overbite	Grouping*	0.04	0.35	0.66–0.74	0.91	0.21	0.38	–0.54 to 0.95	0.58	0.11	0.31	–0.51 to 0.74	0.72
	Baseline	-	-	-	-	-	-	-	-	0.44	0.09	0.27–0.61	<0.001
	R^2^ / R^2^ adjusted	0.001 / –0.010				0.003 / –0.007				0.211 / 0.194			
Mx1H	Grouping*	–0.23	0.16	–0.55 to 0.09	0.15	–0.20	0.17	–0.54 to 0.14	0.26	–0.22	0.10	–0.41 to –0.02	0.03
	Baseline	-	-	-	-	-	-	-	-	0.48	0.04	0.40–0.55	<0.001
	R^2^ / R^2^ adjusted	0.021 / 0.011				0.013 / 0.003				0.644 / 0.636			
Mx6H	Grouping*	–0.18	0.10	–0.38 to 0.02	0.08	0.06	0.06	–0.06 to 0.19	0.32	0	0.06	–0.11 to 0.11	0.97
	Baseline	-	-	-	-	-	-	-	-	0.73	0.05	0.64–0.83	<0.001
	R^2^ / R^2^ adjusted	0.032 / 0.022				0.010 / 0.000				0.720 / 0.714			
MxPeri	Grouping*	1.19	0.65	–0.10 to 2.48	0.07	–0.09	0.29	–0.66 to 0.48	0.75	0.12	0.27	–0.41 to 0.66	0.65
	Baseline	-	-	-	-	-	-	-	-	0.83	0.04	0.76–0.91	<0.001
	R^2^ / R^2^ adjusted	0.033 / 0.023				0.001 / –0.001				0.842 / 0.839			

b, unstandardized regression coefficient; CI, confidence interval; Mx1H, maxillary central incisor crown height; Mx6H, maxillary first molar crown height; MxPeri, maxillary dental arch perimeter; SE, standard error.

* G2 expressed with reference to G1.

Table 2 illustrates important differences among the three methods, which can be seen in terms of the following: (1) effect estimate (seen by the unstandardised effect *b*); (2) the resulting *P* values; (3) the precision of the statistical estimates (seen by the standard errors [SEs]); and (4) the R^2^ values, which are typically interpreted as the % of the outcome variability ‘explained’ by the model. It is obvious from this table that analysing either the end-of-study values (T2) or the before-and-after increment (T1–T2) does not change very much in terms of precision (SE) or R^2^ values. However, analysis of the end-of-study value (T2) adjusting for baseline (T1) in all cases results in considerably more precise results (smaller SEs) and models that fit considerably better to the data (higher R^2^ values). In addition, in many cases we see completely contradicting results from the three analyses. For example, the effect of treatment on the crown height of the upper central incisor (Mx1H) is not statistically significant when using end-of-study values (*P* = 0.15) or before-and-after increments (*P* = 0.26) but has a *P* = 0.03 when using end-of-study values adjusted for baseline. The same can be seen for crown height of the upper first molar (Mx6H) and the maxillary arch perimeter (MxPeri) that have *P* values that are often interpreted by some authors wrongly as statistical trends (*P* values of 0.08 or 0.07) in the analysis of end-of-study values but get considerably higher *P* values with the latter, more precise analysis also adjusting for baseline (*P* values of 0.97 and 0.65). Therefore, it can be pretty obvious that the latter approach might have considerable advantages over the other two (and is actually what the authors of the initial trials used to analyse their data). And in any case, it is obvious from Table 2 that statement (C) is also wrong.
